# East Tennessee Medical Society

**Published:** 1846-08

**Authors:** 


### EAST TENNESSEE MEDICAL SOCIETY.

We have received from Dr. Ramsey an excellent essay by Professor Dickson *on Quinine*, read before the Medical Society of East Tennessee at its meeting in May, which will appear in our next number. This was the second annual meeting of this Society, and was spirited and interesting. We hope to receive fuller accounts of its future meetings. The officers of the Society for the ensuing year are, J. B. Reese, President; S. B. Bowls and W. F. Barr, Vice Presidents; F. A. Ramsey, Corresponding Secretary; R. B. Strong, Recording Secretary; Wm. Rodgers, Treasurer.

